# Clinical response beyond the Systemic Lupus Erythematosus Responder Index: post-hoc analysis of the BLISS-SC study

**DOI:** 10.1136/lupus-2018-000288

**Published:** 2018-11-26

**Authors:** Ronald F van Vollenhoven, William Stohl, Richard A Furie, Norma Lynn Fox, James G Groark, Damon Bass, Milena Kurtinecz, Bonnie F Pobiner, William J Eastman, Tania Gonzalez‑Rivera, David Gordon

**Affiliations:** 1 Department of Rheumatology, Amsterdam Rheumatology and Immunology Center ARC, Amsterdam, The Netherlands; 2 University of Southern California Keck School of Medicine, Los Angeles, California, USA; 3 Northwell Health, Great Neck, New York, USA; 4 GSK, Clinical Development – Immuno-Inflammation, Rockville, Maryland, USA (at the time of the study); 5 GSK, Clinical Development – Immuno-Inflammation, Philadelphia, Pennsylvania, USA; 6 GSK, Immuno-Inflammation and Future Pipeline, Collegeville, Pennsylvania, USA; 7 GSK, Biostatistics, Immuno-Inflammation, Collegeville, Pennsylvania, USA; 8 GSK, Medical Affairs, Research Triangle Park, North Carolina, USA; 9 GSK, Medical Affairs – Immuno-Inflammation, Research Triangle Park, North Carolina, USA (at the time of the study); 10 GSK, Medical Affairs, Rockville, Maryland, USA

**Keywords:** belimumab, corticosteroids, FACIT-fatigue, systemic lupus erythematosus, SLE responder index

## Abstract

**Objective:**

The Systemic Lupus Erythematosus (SLE) Responder Index (SRI), developed as a primary outcome measure for use in clinical trials, captures improvement in SLE disease activity without concomitant worsening in disease manifestations. This study investigated the relationships between the SRI and clinical/laboratory correlates of SRI response in patients with SLE.

**Methods:**

This was a post-hoc analysis of the phase III, double-blind, placebo-controlled study of subcutaneous BeLimumab in Subjects with Systemic lupus erythematosus - SubCutaneous (BLISS-SC). Patients were randomised to weekly belimumab 200 mg subcutaneously or placebo, plus standard SLE therapy. Changes from baseline to week 52 in clinical and laboratory parameters were compared among SRI responders and non-responders, irrespective of the treatment received.

**Results:**

SRI responders (n=475) had significantly better (p<0.0001) outcomes compared with non-responders (n=358), including (by definition) higher proportions achieving ≥4-point improvement in Safety of Estrogens in Lupus Erythematosus National Assessment-SLE Disease Activity Index (100.0% vs 2.0%), no worsening in British Isles Lupus Assessment Group (BILAG; 0 new BILAG A or ≤1 new BILAG B score; 100.0 % vs 50.3%) and no worsening (<0.3-point increase) in Physician’s Global Assessment score (100.0% vs 49.7%). Among patients receiving >7.5  mg/day corticosteroids at baseline, significantly more SRI responders had reductions in prednisone dose to ≤7.5 mg/day than non-responders. SRI responders reported lower flare rates and improvements in serological markers and Functional Assessment of Chronic Illness Therapy-Fatigue score than non-responders.

**Conclusion:**

SRI response is associated with improvements in clinical and laboratory measures, strengthening its value as a clinically meaningful primary endpoint in clinical trials.

## Introduction

Systemic lupus erythematosus (SLE) is a chronic autoimmune disease that is highly heterogeneous in its presentation.^1^ Accurate clinical assessment of SLE is imperative due to its diverse presentation and the fluctuations in disease symptoms and severity over time.^2^ Most SLE-specific measures of disease activity are used only in clinical trials and observational studies in specialised centres; they are not routinely used in everyday clinical practice, as they are not considered practical for busy clinical settings.^3^

The Systemic Lupus Erythematosus Responder Index (SRI) was developed as a primary outcome measure for use in clinical trials.^4^ It uses the Safety of Estrogens in Lupus Erythematosus National Assessment-SLE Disease Activity Index (SELENA-SLEDAI), British Isles Lupus Assessment Group (BILAG), and the Physician’s Global Assessment (PGA) to measure changes in disease activity in patients with SLE.^4^ The SRI was designed to capture improvement in SLE disease activity without concomitant worsening in other disease manifestations.^4^ However, the perceived complexity of the SRI suggests its clinical meaningfulness in relation to measurements of SLE disease activity routinely used in ‘real-world’ clinical practice requires clarifying.

A post-hoc analysis of the phase III trials of intravenous belimumab (BLISS-52 and BLISS-76) assessed the association of the SRI response at week 52 with clinical and laboratory measures of SLE, patient-reported health-related quality of life (HRQoL) and fatigue among SRI responders and non-responders, irrespective of treatment. The results of this study suggested that an SRI response is associated with improved clinical, laboratory and patient-reported outcome measures in patients with SLE.^5^

BLISS-SC (BEL112341; NCT01484496), a phase III, randomised, 52-week trial was conducted to assess the efficacy and safety of subcutaneous belimumab in patients with SLE.^6^ In this post-hoc analysis, we investigated the relationships between SRI response and clinical and laboratory measures of SLE among patients in the BLISS-SC study to examine the clinical relevance of achieving an SRI response.

## Materials and methods

### Study design and patients

This was a post-hoc analysis of the phase III, multicentre, 52-week, placebo-controlled BLISS-SC study (BEL112341; NCT01484496), carried out at 207 sites in 31 countries, between November 2011 and September 2015.^6^

The study design and methods have been described in detail previously^6^ and are summarised here. Patients were required to have a diagnosis of SLE according to the American College of Rheumatology criteria,^7^ with positive antinuclear antibodies (ANA) and/or anti-double-stranded DNA (anti-dsDNA), a SELENA-SLEDAI score ≥8, and to be receiving stable standard of care therapy (SoC) for ≥30 days. Patients with severe lupus kidney disease (proteinuria >6 g/24 hours or equivalent using spot urine protein to creatinine ratio, or serum creatinine >2.5 mg/dL), severe active nephritis (requiring acute therapy not permitted in the study protocol (eg, intravenous cyclophosphamide), or have required haemodialysis, or high-dose prednisone or equivalent within 90 days prior to baseline), or severe central nervous system (CNS) lupus (including seizures, psychosis, organic brain syndrome, cerebrovascular accident, cerebritis or CNS vasculitis) requiring therapeutic intervention within 60 days of baseline were excluded. Patients were randomised 2:1 to weekly belimumab 200 mg subcutaneously or placebo administered using a prefilled syringe, plus SoC. The primary endpoint of BLISS-SC was the SRI response rate at week 52. An SRI responder was defined as a patient who had ≥4-point reduction in SELENA-SLEDAI score, no new BILAG A or ≤1 new BILAG B domain scores, and no deterioration (<0.3-point increase) from baseline in the PGA.^4^ Severe flare rates, reductions or increases in prednisone use, and changes in fatigue, as measured by the Functional Assessment of Chronic Illness Therapy (FACIT)-Fatigue scale, are not included within the SRI, but were included as additional endpoints. Patients were considered non-responders if they did not meet the SRI response criteria, withdrew before week 52 or received protocol-prohibited medications.

All patients provided written informed consent prior to enrolment. The study was conducted in accordance with the Declaration of Helsinki 2008.^8^

### Endpoints and assessments

Changes from baseline to week 52 in clinical and laboratory parameters among SRI responders and non-responders were compared. Parameters analysed included change from baseline to week 52 in SELENA-SLEDAI, BILAG, PGA and corticosteroid use (prednisone equivalent dose). The incidence of any (mild, moderate or severe) Systemic Lupus Erythematosus Flare Index (SFI) and severe SFI flares were also compared. Health outcomes were assessed using FACIT-Fatigue scores.^9 10^ Biomarker assessments included normalisation of anti-dsDNA, and serum complement levels (C3 and C4) and mean per cent change from baseline in B cell subsets (CD20+; naïve CD19+/CD20+/CD27–; activated CD19+/CD20+/CD69+; memory CD19+/CD20+/CD27+; plasmacytoid CD19+/CD20+/CD138+; plasma CD19+/CD20–/CD138+; SLE subset CD19+/CD38b+/CD27b+ lymph; transitional CD19+/CD24b+/CD38b+/CD27–). B cell counts (with the exception of CD20+ B cells) were normalised to CD19+ B cell counts.

### Data analyses

In this post-hoc analysis, SRI responders and non-responders were compared regardless of the treatment received. With the exception of the biomarker analyses, all analyses were conducted using the last observation carried forward.

Components of the SRI and changes in corticosteroid dose from baseline to week 52 were compared using logistic regression models with a standard least squares (LS) method. Changes in SELENA-SLEDAI, BILAG, PGA, corticosteroid dose, FACIT-Fatigue and biomarkers were assessed using an analysis of covariance model comparing SRI responders and non-responders at week 52. The time to first severe SFI flare was compared between SRI responders and non-responders using a Cox proportional hazards model. Shifts in anti-dsDNA and complement levels were evaluated using Fisher’s exact test.

The analyses were performed using Statistical Analysis Software (SAS) V.9.3.

## Results

### Patient population

The intent-to-treat population comprised 836 patients; 3 patients did not have a PGA assessment at baseline, so they could not be categorised as SRI responders or non-responders and were therefore excluded from these post-hoc analyses. In total there were 475 SRI responders and 358 SRI non-responders. Overall, 677 (81.0%) patients completed the study, and 159 (19.0%) patients withdrew prior to week 52; common reasons for withdrawal were adverse events (7.8%) and patient request (3.2%). Baseline characteristics were balanced across SRI responders and non-responders (table 1). The majority of patients were female (94.4%), and the mean (standard deviation [SD]) age was 38.6 (12.28) years. The mean (SD) SELENA-SLEDAI scores at baseline were 10.9 (3.08; SRI responders) and 9.8 (3.09; SRI non-responders) (table 1).

**Table 1:** Patient demographics and characteristics

ITT population	SRI non-responders	SRI responders	Overall
	(n=358)	(n=475)	(n=833)
Female, n (%)	335 (93.6)	451 (94.9)	786 (94.4)
Mean age, years (SD)	39.1 (12.65)	38.1 (11.98)	38.6 (12.28)
Median SLE disease duration, years (range)	5.0 (0–38)	4.0 (0–33)	4.4 (0–38)
Mean baseline SELENA-SLEDAI score (SD)	9.8 (3.09)	10.9 (3.08)	10.4 (3.14)
SELENA-SLEDAI score ≤9, n (%)	169 (47.2)	144 (30.3)	313 (37.6)
SELENA-SLEDAI score ≥10, n (%)	189 (52.8)	331 (69.7)	520 (62.4)
BILAG organ domain involvement*, n (%)			
≥1A or 2B	246 (68.7)	351 (73.9)	597 (71.7)
≥1A	61 (17.0)	77 (16.2)	138 (16.6)
≥1B	325 (90.8)	429 (90.3)	754 (90.5)
No A or B	19 (5.3)	23 (4.8)	42 (5.0)
PGA, mean (SD)	1.6 (0.46)	1.6 (0.42)	1.6 (0.44)
Prednisone, n (%)			
0 mg/day	59 (16.5)	54 (11.4)	113 (13.6)
>0 to ≤7.5 mg/day	85 (23.7)	133 (28.0)	218 (26.2)
>7.5 mg/day	214 (59.8)	288 (60.6)	502 (60.3)
≥1 SFI flare, n (%)†	66 (18.4)	83 (17.5)	149 (17.9)
≥1 severe SFI flare, n (%)†	8 (2.2)	4 (0.8)	12 (1.4)
Anti-dsDNA-positive (≥30 IU/mL), n (%)	250 (69.8)	345 (72.6)	595 (71.4)
Low C3 (<90 mg/dL), n (%)	144 (40.2)	210 (44.2)	354 (42.5)
Low C4 (<10 mg/dL), n (%)	94 (26.3)	121 (25.5)	215 (25.8)
CD20+ B cells median cell count 10^9/L (range)	0.1065 (0.007–1.517)	0.1065 (0.004–1.323)	0.1065 (0.004–1.517)

*Patients may be included in more than one category.

†During the screening period (day −35 to day 0).

Anti-dsDNA, anti-double-stranded DNA; BILAG, British Isles Lupus Assessment Group;ITT, intent-to-treat (all randomised patients treated with ≥1 dose of study treatment); PGA, Physician’s Global Assessment;SELENA-SLEDAI, Safety of Estrogens in Lupus Erythematosus National Assessment-Systemic Lupus Erythematosus Disease Activity Index;SFI, Systemic Lupus Erythematosus Flare Index;SRI, Systemic Lupus Erythematosus Responder Index.

### SRI components: SELENA-SLEDAI, BILAG and PGA

**Figure 1:**
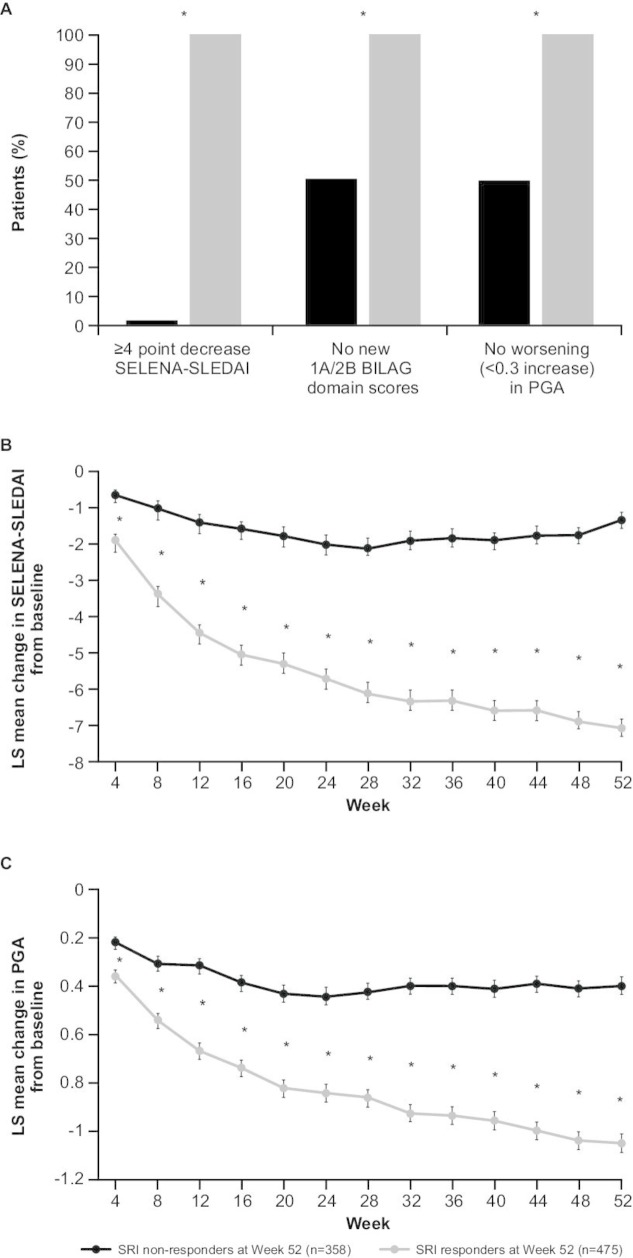
(A) SRI 4 components (week 52); LS mean change from baseline in (B) SELENA-SLEDAI and (C) PGA. *p<0.0001 SRI responders versus non-responders. BILAG, British Isles Lupus Assessment Group; LS, least squares; PGA, Physician’s Global Assessment; SELENA-SLEDAI, Safety of Estrogens in Lupus Erythematosus National Assessment-SLE Disease Activity Index; SRI, Systemic Lupus Erythematosus Responder Index.

At week 52, all SRI responders (by definition) had ≥4-point reduction in SELENA-SLEDAI score compared with 2.0% of non-responders (p<0.0001; (figure 1A). SRI responders consistently had a significantly greater LS mean (standard error [SE]) improvement in SELENA-SLEDAI score than non-responders from baseline to week 52 (week 52: –7.01 (0.225) vs –1.31 (0.217), respectively; p<0.0001) (figure 1B).

Significantly more SRI responders (100%, by definition) had no worsening attributable to SLE in multiple organ systems as measured by BILAG (no new BILAG A or ≤1 new BILAG B score) compared with non-responders (50.3%; p<0.0001) at week 52 (figure 1A). From baseline to week 52, significantly greater improvements were seen in all BILAG and SELENA-SLEDAI organ domains for SRI responders compared with non-responders (table 2).

**Table 2:** Improvement in BILAG (A or B) and SELENA-SLEDAI by organ domain/system

	SRI non-responders (n=358)	SRI responders (n=475)	Treatment difference, SRI responders vs non-responders (95% CI)	P values*
**BILAG organ domain**				
General				
A or B score at baseline†, n	28	40		
Improvement at week 52‡, n (%)	15 (53.6)	39 (97.5)	43.9 (24.83 to 63.02)	<0.0001
Cardiovascular and respiratory				
A or B score at baseline†, n	12	8		
Improvement at week 52‡, n (%)	3 (25.0)	8 (100.0)	75.0 (50.50 to 99.50)	0.0014
Haematology				
A or B score at baseline†, n	67	70		
Improvement at week 52‡, n (%)	12 (17.9)	32 (45.7)	27.8 (12.95 to 42.65)	0.0005
Mucocutaneous				
A or B score at baseline†, n	235	329		
Improvement at week 52‡, n (%)	54 (23.0)	235 (71.4)	48.5 (41.19 to 55.71)	<0.0001
Musculoskeletal				
A or B score at baseline†, n	251	369		
Improvement at week 52‡, n (%)	50 (19.9)	338 (91.6)	71.7 (65.98 to 77.37)	<0.0001
Neurological				
A or B score at baseline†, n	1	3		
Improvement at week 52‡, n %	1 (100.0)	3 (100.0)	0.0 (0.00 to 0.00)	
Renal				
A or B score at baseline†, n	39	44		
Improvement at week 52‡, n (%)	12 (30.8)	37 (84.1)	53.3 (35.25 to 71.39)	<0.0001
Vasculitis				
A or B score at baseline†, n	29	42		
Improvement at week 52‡, n (%)	7 (24.1)	37 (88.1)	63.9 (45.56 to 82.36)	<0.0001
**SELENA-SLEDAI organ system improvement, by category, n (%**)				
CNS total				
Baseline involvement†, n	4	5		
Improvement at week 52§, n %)	0 (0.0)	5 (100.0)	100.0 (100.00 to 100.00)	0.0079
Cardiovascular and respiratory total				
Baseline involvement†, n	19	28		
Improvement at week 52§, n %)	5 (26.3)	25 (89.3)	63.0 (40.09 to 85.85)	<0.0001
Haematological total				
Baseline involvement†, n	27	46		
Improvement at week 52§, n (%)	6 (22.2)	24 (52.2)	30.0 (8.64 to 51.27)	0.0146
Immunological total				
Baseline involvement†, n	267	369		
Improvement at week 52§, n (%)	18 (6.7)	143 (38.8)	32.0 (26.20 to 37.82)	<0.0001
Mucocutaneous total				
Baseline involvement†, n	303	431		
Week 52§, n (%)	92 (30.4)	355 (82.4)	52.0 (45.70 to 58.31)	<0.0001
Musculoskeletal total				
Baseline involvement†, n	256	398		
Improvement at week 52§, n (%)	28 (10.9)	363 (91.2)	80.3 (75.54 to 85.00)	<0.0001

*Fisher’s exact test.

†Number used as denominator for percentages.

‡Patients who have an A at baseline and change to a B, C or D, or patients with a B at baseline who change to a C or D, are considered to have improvement.

§An improvement is defined as a decrease (compared with baseline) in SELENA-SLEDAI score within the same organ system.

BILAG, British Isles Lupus Assessment Group;CNS, central nervous system; SELENA-SLEDAI, Safety of Estrogens in Lupus Erythematosus National Assessment- Systemic Lupus Erythematosus Disease Activity Index; SRI, Systemic Lupus Erythematosus Responder Index.

All SRI responders (by definition) had no worsening (<0.3-point increase) in their overall condition as assessed by the PGA score compared with 49.7% of non-responders (p<0.0001) from baseline to week 52 (figure 1A). SRI responders had a significantly greater LS mean (SE) improvement in PGA score from baseline to week 52 compared with non-responders (−1.02 (0.036) vs −0.39 (0.034), respectively; p<0.0001) (figure 1C).

### Additional components: prednisone use, flares, biomarkers and FACIT-Fatigue

#### Prednisone use

**Figure 2:**
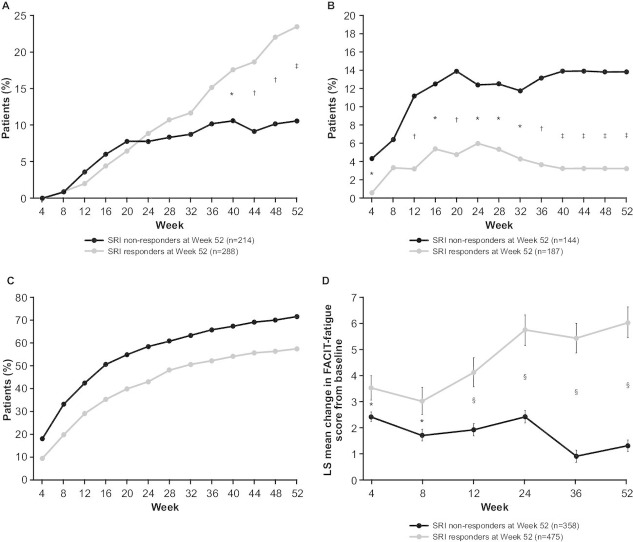
(A) Decrease in prednisone dose from >7.5 mg/day at baseline to ≤7.5 mg/day; (B) increase in prednisone dose from ≤7.5 mg/day at baseline to >7.5 mg/day; (C) cumulative incidence of SFI flares; (D) LS mean change from baseline in FACIT-Fatigue score. *p<0.05; †p<0.01; ‡p<0.001; §p≤0.0001. FACIT-Fatigue, Functional Assessment of Chronic Illness Therapy-Fatigue; LS, least squares; SFI, Systemic Lupus Erythematosus Flare Index; SRI, Systemic Lupus Erythematosus Responder Index.

At baseline, 60.6% of SRI responders and 59.8% of non-responders received >7.5 mg/day prednisone (or equivalent; table 1). Among patients receiving >7.5 mg/day prednisone at baseline, significantly more SRI responders (68/288; 23.6%) were able to reduce prednisone dose to ≤7.5 mg/day at week 52 than non-responders (23/214; 10.7%; odds ratio[OR] 2.49, 95% confidence interval [CI] 1.49 to 4.15; p=0.0005) (figure 2A). There was a significant difference between the two groups from week 40 to week 52. Fewer SRI responders who received prednisone ≤7.5 mg/day at baseline had increases in dose to >7.5 mg/day at week 52 than non-responders (6/187 (3.2%) vs 20/144 (13.9%); OR 0.20, 95% CI 0.08 to 0.52; p=0.0009). There was a significant difference between the two groups from week 4 to week 52, with the exception of week 8 (figure 2B).

#### Flares

Significantly fewer SRI responders (272/475; 57.3%) than non-responders (256/358; 71.5%; p<0.0001) had mild, moderate or severe flares over the 52-week period (figure 2C). The median time (25th, 75th percentile) to first flare was longer in SRI responders (223 (85, non-calculable) days) than non-responders (113 (57, 330) days; p<0.0001). Significantly more SRI responders (459/475; 96.6%) than non-responders (260/353; 73.7%; p<0.0001) had no severe flares; therefore, severe flares occurred in 3.4% of SRI responders vs 26.3% of non-responders.

#### Biomarkers

**Figure 3:**
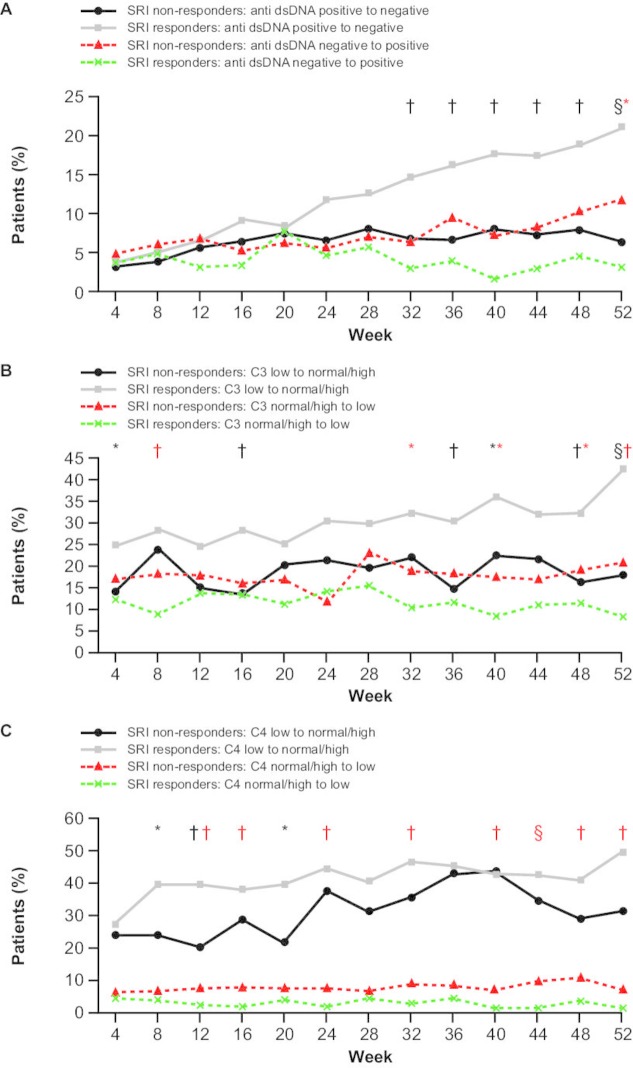
Change in biomarkers over time: (A) anti-dsDNA (IU/mL), (B) C3 (mg/dL) and (C) C4 (mg/dL). Anti-dsDNA, anti-double-stranded DNA; SRI, Systemic Lupus Erythematosus Responder Index. 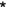
p<0.05 SRI responders versus non-responders (anti-dsDNA positive to negative; C3/C4 low to normal/high); 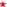
p<0.05 SRI responders versus non-responders (anti-dsDNA negative to positive; C3/C4 normal/high to low); 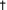
p<0.01 SRI responders versus non-responders (anti-dsDNA positive to negative; C3/C4 low to normal/high); 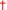
p<0.01 SRI responders versus non-responders (anti-dsDNA negative to positive; C3/C4 normal/high to low); 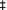
p<0.001 SRI responders versus non-responders (anti-dsDNA positive to negative; C3/C4 low to normal/high); 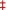
p<0.001 SRI responders versus non-responders (anti-dsDNA negative to positive; C3/C4 normal/high to low); 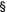
p≤0.0001 SRI responders versus non-responders (anti-dsDNA positive to negative; C3/C4 low to normal/high); 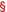
p≤0.0001 SRI responders versus non-responders (anti-dsDNA negative to positive; C3/C4 normal/high to low).

At baseline, 71.4% of patients were anti-dsDNA-positive (≥30 IU/mL), 42.5% had low C3 levels (<90 mg/dL) and 25.8% had low C4 levels (<10 mg/dL) (table 1). Significantly more SRI responders (71/336; 21.1%) than non-responders (9/140; 6.4%) had normalised anti-dsDNA at week 52 (p<0.0001) (figure 3A). The median (25th, 75th percentile) percentage change in anti-dsDNA antibodies was –46.7% (–68.3, –10.7) for SRI responders compared with –16.7% (–49.5, 5.5; p=0.0171) for non-responders, from baseline to week 52. Among patients with low C3 at baseline, a greater proportion of SRI responders shifted to normal/high C3 levels at week 52 (87/205; 42.4%) compared with non-responders (14/78; 17.9%; p<0.0001) (figure 3B). From baseline to week 52, the median (25th, 75th percentile) percentage change in C3 across all patients was 6.3% (–6.8, 21.8) for SRI responders compared with –2.1% (–10.9, 12.2; p=0.0024) for non-responders. In patients with low C4 at baseline, more SRI responders shifted to normal/high levels at week 52 (59/120; 49.2%) compared with non-responders (14/45; 31.1%; p=0.0523) (figure 3C). From baseline to week 52, the median (25th, 75th percentile) percentage change in C4 across all patients was 16.7% (0, 44.4) for SRI responders compared with 5.9% (–14.3, 24.1; p=0.0540) for non-responders.

Similar decreases in the levels of B cell subsets (CD20+; naïve CD19+/CD20+/CD27−; activated CD19+/CD20+/CD69+; memory CD19+/CD20+/CD27+; plasmacytoid CD19+/CD20+/CD138+; plasma CD19+/CD20−/CD138+; SLE subset CD19+/CD38b+/CD27b+ lymph; transitional CD19+/CD24b+/CD38b+/CD27−) were observed in SRI responders and non-responders from baseline to week 52 (data not shown).

### FACIT-Fatigue

The mean (SE) percentage change in FACIT-Fatigue score from baseline to week 52 was greater for SRI responders (35.6% (4.02)) than non-responders (18.7% (3.53); p<0.0001; figure 2D). A significantly higher proportion of SRI responders had a minimal clinically important difference improvement in FACIT-Fatigue score of ≥4 points at week 52 compared with non-responders (53.9% vs 25.3%; p<0.0001).

## Discussion

As SLE is a heterogeneous disease that impacts multiple organ systems,^1 2^ the SRI was designed to distinguish global response from non-response, based not only on improvement in SLE disease activity but on an absence of worsening of disease manifestations.^4^ It has been used in the four key phase III trials of belimumab plus SoC for the treatment of SLE: BLISS-52, BLISS-76, BLISS-SC and the recently completed study of intravenous belimumab in North East Asia.^6 11–13^ In all four trials the proportions of patients with an SRI response were significantly higher in the belimumab group compared with placebo.^6 11 12^ Furthermore, a post-hoc analysis of the BLISS-52 and BLISS-76 trials demonstrated that SRI responders had greater improvements in clinical, laboratory and HRQoL measures compared with non-responders.^5^ In this post-hoc analysis of data from the BLISS-SC study, we investigated the relationships between SRI response and clinical and laboratory measures of SLE that are more commonly used in routine practice in order to examine the clinical meaningfulness of SRI response.

Overall, SRI responders had statistically significant better outcomes in multiple parameters compared with non-responders, including improvements in the individual SRI components (SELENA-SLEDAI, BILAG and PGA scores). These improvements observed within the individual SRI components demonstrate how the simpler individual components correlate effectively with the more complex SRI composite index. Reductions were also observed in non-SRI components, including reductions in corticosteroid dose, lower flare rates, improvements in serological markers and improvements in FACIT-Fatigue score. Improvement in these non-SRI components demonstrates the relevance of the SRI, an index routinely used within clinical trials, in reflecting measures that are more commonly used in clinical practice. Our findings are in line with those of a previous post-hoc analysis of the BLISS-52 and BLISS-76 studies of intravenous belimumab that found that SRI responders had greater improvements in clinical, serological and HRQoL measures of SLE disease activity compared with non-responders.^5^ For example, in this study 57.3% of SRI responders and 71.5% of non-responders had SFI flares (p<0.0001), compared with 69.9% and 82.7%, respectively, in the intravenous belimumab post-hoc analysis (p<0.0001).^5^ Both of these studies also demonstrated that SRI responders had clinically significant greater improvements in BILAG and SELENA-SLEDAI organ domains than non-responders.^5^ In this study, greater improvements in all organ domains were observed in SRI responders compared with non-responders; however, it should be noted that in some cases the numbers of patients in the analyses were small.

Many studies have demonstrated that anti-dsDNA antibodies and low complement levels are associated with increased SLE disease severity.^14–18^ In the present study, an SRI response was associated with increases in C3 and/or C4 levels and decreases in anti-dsDNA antibody titres. There were no significant differences in changes in the levels of B cell subsets between SRI responders and non-responders. A pooled analysis of the BLISS-52 and BLISS-76 trials showed that belimumab, compared with placebo, was associated with significant reductions in CD20+ B cells and multiple B cell and plasma cell subsets, including naïve and activated B cells, while preserving the memory B cell subset^19^; however, there were no significant differences in the reductions of B cells in SRI responders versus non-responders.^5^ Similarly, in the current analysis, there were patients in both the SRI responder and non-responder groups who received belimumab, which may explain why there were no significant differences in changes in the levels of B cell subsets between the two groups.

Fatigue is one of the commonly reported symptoms among patients with SLE, and it considerably impacts patients’ lives.^20^ The mean improvements in FACIT-Fatigue scores reported by SRI responders were greater than in non-responders (35.6% vs 8.7%, respectively). The changes from baseline exceeded the minimal clinically important difference of ≥4 points^9 10^ at week 52 in over twice as many SRI responders compared with non-responders (53.9% vs 25.3%, respectively).

Interpretation of these results is limited by the post-hoc and observational nature of the analyses. Examination of the clinical trial population based on the primary endpoint eliminates the randomised balance of baseline characteristics in the treatment groups. Further, the inclusion criteria did not permit inclusion of patients with SELENA-SLEDAI scores <8 at screening, nor did they permit entry of patients with severe lupus kidney disease, severe active nephritis or active CNS lupus disease; thus, no conclusions can be made about these subgroups. Despite these limitations, the study used a large and robust data set to demonstrate the significance of an SRI response in terms that are relevant to clinicians.

In conclusion, patients who were SRI responders, regardless of treatment, demonstrated improvements in numerous clinical and serological measures of disease activity compared with non-responders. This post-hoc analysis provides evidence that the SRI response represents a clinically meaningful outcome that can be used during clinical trials for patients with active, autoantibody-positive SLE.
